# The abundance and localization of heat shock proteins (HSP)-60, -70, and -90 in the oviductal ampulla of hamadryas baboon (*Papio hamadryas*) during the menstrual cycle

**DOI:** 10.1007/s11259-023-10270-3

**Published:** 2023-12-01

**Authors:** Maria Albrizio, Salvatore Desantis, Luca Lacitignola, Pietro Laricchiuta, Antonio Ciro Guaricci, Mario Cinone

**Affiliations:** 1 Department of Precision and Regenerative Medicine and Jonian Area (DiMePRe-J), University of Bari-Aldo Moro, S.P. 62 Casamassima Km 3, Valenzano, 70010 Italy; 2 Safari Zoo, Fasano, 72015 Italy

**Keywords:** Fallopian tubes, Primates, Sex hormones, Vaginal cytology, Salpingectomy

## Abstract

The presence of HSPs in female reproductive and their relationship with the steroid hormone fluctuation have been reported in several mammals but not in non-human primates. The present research dealt with the oviductal expression and localization of the more studied HSPs (60, 70, and 90) as well as the morphological changes in the Hamadryas baboon (*Papio hamadryas*) during the follicular, preovulatory, and luteal phases of the menstrual cycle. Therefore, western blots, histomorphological, and immunohistochemical analyses were carried out. The results of western blot analysis displayed the lowest HSP expression in the luteal phase. The histomorphology showed that the mucosal epithelium consisted of undifferentiated cuboidal cells in follicular and luteal phases and well-distinguishable columnar ciliated and non-ciliated cells during the preovulatory phase. Immunohistochemistry evidenced that the mucosal epithelium contained cytoplasmic and nuclear HSP60, 70, and 90 immunostaining in the follicular and luteal phases. During the preovulatory phase, the non-ciliated cells showed: (i) cytoplasmic HSP60; (ii) nuclear and cytoplasmic HSP90. Ciliated cells showed cytoplasmic and ciliary HSP70 and ciliary HSP90. The stromal cells and myocytes of muscular layer displayed a decreased cytoplasmic HSP60 in the preovulatory phase and nuclear and low cytoplasmic HSP70 throughout the menstrual cycle. Nuclear HSP90 decreased in ampulla stromal cells and the follicular phase myocytes. These findings indicate that the expression pattern of HSP60,70, and 90 is related to the morphofunctional features of the baboon oviductal ampulla during the menstrual cycle and could represent a referent point for further studies in the oviduct of Primates.

## Introduction

The oviduct is a highly specialized structure of the female reproductive system that assumes one of the most basic roles in the reproductive process, being the site of fertilization, pre-implantation, and embryo development (Li and Winuthayanon 2017). Based on the macroscopic and microscopic features, the oviduct can be divided into four main regions: fimbria, infundibulum, ampulla, and isthmus. Each segment has a specific structure subordinated to the function in charge (Abe 1996; Barton et al. 2020). Particularly, the ampulla is the site where fertilization occurs. The oviduct is a small, elongated, and tubular structure that connects the ovary to the uterus and is formed by a fibromuscular complex composed of layers such as mucosa, smooth muscle layer, and connective serosa.

The important role of the oviduct is entrusted to the luminal fluid that interacts successively with gametes and embryos (Li and Winuthayanon 2017). The oviductal fluid is a complex solution containing ions, energy substrates, amino acids, prostaglandins, steroid hormones, growth factors, and various proteins. The most abundant proteins in oviduct secretions include serum albumin, oviductin (OVGP1), and heat shock proteins (HSPs) (Saint-Dizier et al. 2020).

Heat shock proteins (HSPs) are ubiquitous proteins, which were discovered by Ritossa (1962) and fully characterized in their genetic structure and activity at the end of the last century (Hu et al. 2022). Heat shock proteins are the most conserved proteins in nature (Neuer et al. 2000) and are suddenly expressed upon heat stress that, on the contrary, usually causes a drastic switch-off in the protein expression. Their presence is crucial for cell survival because they are involved not only in protecting the cell from heat shock but also in regulating intracellular transport, maintaining proteins active, in regulating their folding and degradation (Hu et al. 2022). In addition, HSPs can behave as extracellular stress proteins (Calderwood et al. 2007). According to their molecular weight, HSPs are categorized into six families: HSP100, HSP90, HSP70, HSP60, HSP40, and small (15 to 30 kDa) HSPs. Each family of HSPs is composed of members expressed either constitutively or regulated inductively (Schmitt et al. 2007). In animals, the most studied HSPs are those with molecular masses of 60, 70, and 90 kDa (Hu et al. 2022). The HSP60 is mainly contained in the mitochondrial matrix, where it is necessary for the correct folding of mitochondrial proteins and the proteolytic degradation of misfolded or denatured proteins (Khalil et al. 2011; Malik and Lone 2021; Hu et al. 2022). The expression of HSP70 is low under physiological conditions, permitting constitutive cellular activities to proceed, while different stresses strongly induce it (Qian et al. 2006; Hu et al. 2022). In unstressed cells, HSP90 can constitute up to 1% of total cellular proteins (Kuhn et al. 2006). It has a role in maintaining protein conformation under normal conditions by associating with several intracellular proteins such as some receptors, calmodulin, actin, tubulin, and several kinases (Kopecek et al. 2001; Takaki et al. 2007; Sima and Richter 2018; Hu et al. 2022).

The appearance of the menstrual cycle and the modulation of the oviductal morphology and function are regulated by sex hormone fluctuations (Barton et al. 2020) that are also modulators of the HSPs expression in several tissues such as the porcine ovary (Sirotkin and Bauer 2011) and rat hypothalamus (Olazabal et al. 1992). As for the oviduct, sex hormone-depending HSPs expression has been reported in ewes (Soleihavoup et al. 2016), rats (Mariani et al. 2000), buffalos (Kalyanaraman et al. 2022), and bovines (Bauersachs et al. 2004). Proteomic analyses of oviducts revealed the presence of several HSPs in the luminal fluid of cows (Lamy et al. 2016), rabbits (Yu et al. 2019), and ewes (Soleihavoup et al. 2016), whereas immunohistochemical investigations localized the site of the expression of HSPs in cattle (Boilard et al. 2004)], rats (Mariani et al. 2000), and women (Lachance et al. 2007). The role of HSPs in the oviduct is not clearly understood, although it has been demonstrated that HSP60 expressed by oviductal epithelial cells modulates sperm motility in humans (Lachance et al. 2007). Moreover, the members of the Hsp70 family, which are expressed in oviductal cells, increase sperm viability in sows and cattle (Elliott et al. 2009) and participate in the zona pellucida-sperm interaction controlling polyspermy in cattle (Sakatani et al. 2015).

The studies on the localization of HSPs in mammalian oviducts are few and not exhaustive. HSP70 and HSP25 have been detected in the ampulla of rats during the estrous cycle (Mariani et al. 2020). HSP60 has been localized in the ampulla of early estrus cows (Boilard et al. 2004) and women (Lachance et al. 2007). To our knowledge, no reports have been conducted on the presence and localization of HSPs in the oviduct of non-human primates. Baboons, as opposed to laboratory animals, can be considered a beneficial model for translational medicine, being very close to humans in size, reproductive tracts, and menstrual cycle (Bauer 2015). Thus, this study aimed to examine the abundance and localization of the major HSP families (HSP60,70, and 90) in the oviduct of a non-human primate such as the baboon *Papio hamadrya*s (Linnaeus, 1758) throughout the menstrual cycle. Particularly, we dealt with the ampulla because it is the site of oocyte fertilization and early embryonic development.

## Materials and methods

All chemicals were purchased from Sigma-Aldrich (Milano, Italy) unless otherwise stated.

### Animals

In this study, fourteen captive females of *Papio hamadryas* housed in the Safari Zoo (Fasano (BR), South Italy) were enrolled. The subjects were all adults, sexually mature, not pregnant, and without endometrial disorders (hyperplasia, endometritis, neoplasia) evaluated by ultrasonography. All surgical interventions were conducted following Italian law in respect of animal welfare.

The research was performed on oviducts removed during a laparoscopic salpingectomy program for birth control of *Papio hamadryas*. The clinical project was required and authorized with written informed consent by the Zoo’s property (Leo 3000 S.p.a, c/o Safari Zoo) and approved by the Ethical Committee of the Department of Emergency and Organs Transplantation of the University of Bari-Aldo Moro (approval number: 05/2020).

### Laparoscopic salpingectomy

Abdominal ultrasonography was performed before surgery to evaluate the reproductive tract and exclude pregnant females. Animals were kept fasting for 15 h and water was withheld 8 h before surgery. Restrained in a squeeze cage, animals were hand injected with Tiletamine-zolazepam 2.5 mg/kg and Medetomidine 0.03 mg/kg. A 21G intravenous cannula was placed in the cephalic vein, and propofol was administered to allow intubation. During the surgical procedure, animals were maintained with isofluorane. The administration of methadone 0.1 mg/kg IM and meloxicam 0.21 mg/kg as a single dose was necessary to guarantee analgesia. A previous report described the surgical procedures in detail (Lacitignola et al. 2022). Briefly, the 3port technique with 5 mm instruments and a telescope placed at the umbilical and hypogastric regions were employed. The dissection of the oviduct, from the fimbriae to the uterine attachment, was performed with a radiofrequency bipolar vessel-sealing device. Immediately after excision, small fragments of the ampulla were frozen in liquid nitrogen for molecular analysis; the remaining tissue was fixed in 4% (w/v) phosphate-buffered (PBS) paraformaldehyde and processed for histological investigations.

### Identification of the menstrual cycle phases

The baboon reproductive cycle’s phase was identified as previously described (Desantis et al. 2022). Briefly, serum samples obtained by blood centrifugation were used to evaluate beta-estradiol and progesterone concentrations by commercial ELISA kits (monkey ELISA kits, My BioSource; San Diego, CA USA). Measurements were made in duplicate. Moreover, for each subject, the stage of the menstrual cycle was confirmed by evaluating the specific pattern of the vaginal cytology as previously published (Desantis et al. 2022) and here briefly described. A vaginal swab was employed to collect epithelial cells from the anterior vagina. Cells were transferred onto a microscope slide by rolling the swab on it. After fixation, cells were stained by the Diff Quick method and observed under the optical microscope Nikon Eclipse E600 (Nikon, Japan). At least 100 epithelial cells were observed and counted for the presence of epithelial nucleated (parabasal, intermediate, superficial cells) and anucleated (cornified) cells. The presence of leukocytes, erythrocytes and bacteria was also evaluated. The cornification index (CI) was calculated as the number of cornified cells x100/total number of epithelial cells. A CI value > 80% was associated to the preovulatory phase.

### Western blot analysis

Frozen fragments from the oviductal ampulla were pulverized in liquid nitrogen and completely homogenized in cold lysis buffer (PBS, 0.1% Triton-X) in the presence of protease inhibitors as previously described (Desantis et al. 2010). Forty micrograms of proteins were loaded into a precast 4–20% polyacrylamide gel (Criterion, Bio-Rad, Milano, Italy), electrophoresed, and transferred to a PVDF membrane (Millipore, Milano, Italy) by the Trans-Blot®SD Semi-Dry Transfer cell (Bio-Rad, Milano, Italy).

The membrane was hybridized with a solution containing 1 µg of the primary antibody by the Snap i.d. system (Merck-Millipore, Germany). For the identification of HSP60, 70, and 90, the following mouse monoclonal antibodies were employed: anti-HSP 60 (dilution: 1:600 sc-13115, Santa Cruz Biotechnology Inc., USA), anti-HSP 70 (dilution: 1:600 sc-7298, Santa Cruz Biotechnology Inc., USA), and anti-HSP 90 (dilution: 1:600 SMC-107, StressMarq Biosciences Inc., Cadboro Bay, Victoria, Canada). For loading control and normalization, the membrane was also hybridized to rabbit anti-beta-actin antibody (dilution: 1:1500 A2103, SIGMA-Aldrich, USA). The hybridization signal was evidenced by the Vectastain elite system (Vector-Laboratories, Burlingame, CA, USA) following the procedure suggested by the company. In the negative control tests, the hybridization procedure was performed: (a) omitting the primary antibodies and (b) hybridizing the membrane with a solution containing the primary antibodies of the three HSPs let to react with a molar excess of their control peptides (HSP60 control peptide sc-13115P; HSP70 control peptide sc-7298P; HSP90 control peptide SPR102A) and the anti-beta-actin primary antibody. The latter control was also performed to verify the specificity of the obtained signals in this species. Anyway, because all primary antibodies employed were raised against proteins of human origin, a theoretical identity between human and non-human primates was checked. For each HSP, the gene sequences of the two species were aligned by the software “BLAST” (NCBI). Sequence identities were as follows: HSP60 98.20% (sequences accession numbers: M34664 (human), MW981594 (nonhuman primate); HSP70 98.18% (sequences accession numbers: M11717 (human), MW981595 (non-human primate); HSP90 92.58% (sequences accession numbers: AF275719 (human), XR_167694.3 (nonhuman primate). The optical density of each protein band was quantified using Quantity One software (BioRad, Milano, Italy). The values expressed as arbitrary units (A.U.) are presented as the ratio of the specific protein to the corresponding beta-actin optical density. The western blot procedure was repeated twice.

### Histology

#### Tissue preparation

Oviducts from follicular (n = 5), preovulatory (n = 4), and luteal (n = 5) phases of baboon *Papio hamadryas* immediately after salpingectomy were immersed in 4% (w/v) PBS- paraformaldehyde and fixed for 24 h at room temperature (RT). After trimming excess tissue, the ampulla was separated from each oviduct, dehydrated in an ethanol series, cleared in xylene, and embedded in paraffin. Serial Sect. (5 μm thick) were cut and, after de-waxing with xylene and hydration in an ethanol series of descending concentrations, were stained with hematoxylin-eosin for morphological analysis and by immunohistochemistry to reveal the presence and localization of HSPs.

#### Immunohistochemistry

Immunoperoxidase reaction was performed on serial paraffin Sect. (5 μm) using the same mouse monoclonal primary antibodies (1:200) against HSP60, HSP70, or HSP90 used for the western blot procedure. Sections were pretreated (15 min) with 3% H_2_O_2_ in methanol to inhibit endogenous peroxidase activity, then rinsed with PBS and blocked for 30 min with normal horse serum (NHS) (Vector-Laboratories, Burlingame, CA, USA). Sections were incubated overnight at 4 °C in a solution containing anti-HSP60, anti-HSP70, or anti-HSP90 in PBS containing 2.5% NHS. After rinsing in PBS, sections were incubated with Biotinylated Universal Antibody (Vector-Laboratories, Burlingame, CA, USA). Sections were again rinsed in PBS, and the staining was visualized by incubating in DAB (Vector-Laboratories) solution for 7 min. The specificity of immunohistochemical staining was tested by replacing either the primary antibodies, Biotinylated Universal Antibody, or the ABC complex with PBS or by incubating sections with a solution containing the primary antibody absorbed with a molar excess of its control peptide under these conditions, staining was abolished.

The evaluation of staining intensities was based on subjective estimates of three of the authors (S.D., M.A., M.C.), and the inter- and intraobserver error was tested to assess the reproducibility of the system. A high degree of consistency was found among observers.

### Statistical analysis

The normal distribution of the quantitative HSP data was assessed by the Kolmogorov-Smirnov test. The amounts of HSPs among the different phases of the reproductive cycle were analyzed by the non- parametric Kruskal-Wallis’ test and Mann-Whitney U-test, when appropriate and values expressed in arbitrary units (A.U.) by a bar graphic as mean ± standard deviation (S.D.). P values < 0.05 were considered significant.

## Results

### The abundance of HSP proteins in the ampulla oviduct of adult hamadryas baboons during the menstrual cycle

**Fig. 1:**
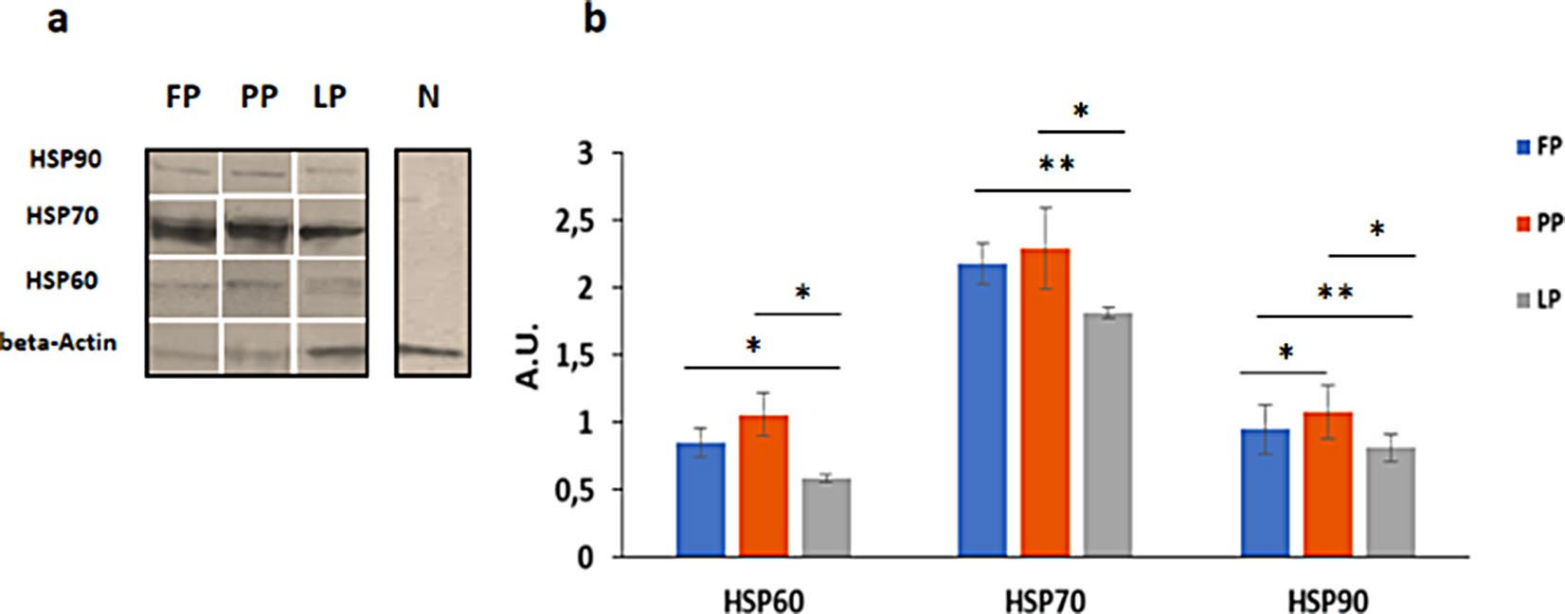
Western blot analysis of HSP 60, 70, and 90 proteins in the *Papio hamadryas* oviductal ampulla during the menstrual cycle. Panel a: Representative immunoblot images of the three HSPs and beta-actin in the ampulla oviduct samples. Beta-actin protein was used as an internal control. Panel b: The bar graphic represents the densitometric analysis of the three HSPs. The relative intensity was determined by the ratio of each HSP to its corresponding internal control as measured by densitometry. A.U., arbitrary units; LP, luteal phase; FP, follicular phase; PP, preovulatory phase, N negative control. *= P < 0.05; **= P < 0.01 determined by the non-parametric Kluskal- Wallis’ test and Mann-Whitney U-test, when appropriate. Data are represented as mean ± standard deviation of four or five baboon tissue samples for preovulatory, follicular and luteal phases. Bars, standard deviation; asterisks indicate significant differences between phases

The quantitative change in the ampulla HSPs amount was evaluated by Western blot analysis. As shown in Fig. 1- panel a, HSP60, 70, and 90 were expressed in the *Papio hamadryas* oviduct during the follicular, preovulatory, and luteal phases. The absence of positive signals for HSPs in the negative control (Fig. 1, lane N) demonstrates the specificity of the employed anti-HSP primary antibodies.

The densitometric analysis represented as a bar chart in panel b, shows that all the three HSPs were expressed at the lowest level in the luteal phase. Comparing the expression level of each HSP between the follicular phase and the luteal phase, we found statistically significant differences (HSP60, P < 0.05; HSP70 and 90. P < 0.01), and this difference in the protein expression was still evident in the preovulatory phase with respect to the luteal phase (P < 0.05). Only HSP90 expression changed significantly between the follicular and preovulatory phases (P < 0.05). The expression level of the oviductal HSP70 is the highest compared to those of HSP60 and HSP90 (HSP70 vs. HSP60; HSP70 vs. HSP90, P < 0.001).

### Histological analysis

**Fig. 2:**
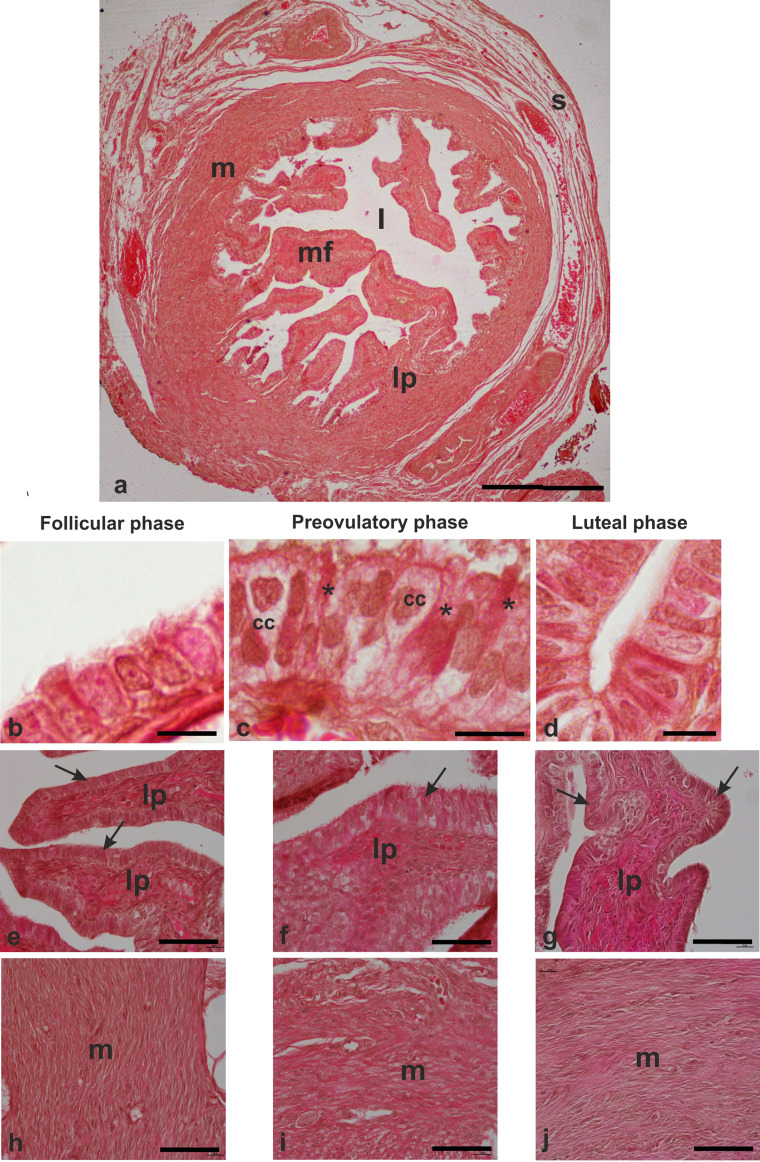
Light micrographs showing histological features of the baboon *Papio hamadryas* oviductal ampulla. **a**, low magnification of a cross-sectioned of ampulla. **b,c,d**, details of morphological features of the ampulla epithelium during the menstrual cycle. **e,f,g**, mucosal folds constituted of covering epithelium and lamina propria. **h-j**, muscular layer. cc, ciliated cell; l, lumen; lp, lamina propria; m, muscularis; mf, mucosal fold; s, serosa; arrow, mucosal epithelium; asterisk, non-ciliated (secretory) cell. Hematoxylin-eosin staining. Scale bars: 2a, 500 μm; b,c,d, 10 μm; e-j, 30 μm

The histological sections showed that the ampulla is constituted of mucosal folds (covering epithelium and lamina propria) (Fig. 2a,e,f,g), a muscular wall (Fig. 2a,h,i,j), and connective serosa (Fig. 2a).

As it has been previously reported (Desantis et al. 2022), the epithelium lining the mucosal folds consisted of cuboidal and undifferentiated cells during the follicular and luteal phases (Fig. 2b,d) and well-differentiated columnar ciliated and non-ciliated cells during the preovulatory phase (Fig. 2c).

### HSPs immunolocalization

**Fig. 3:**
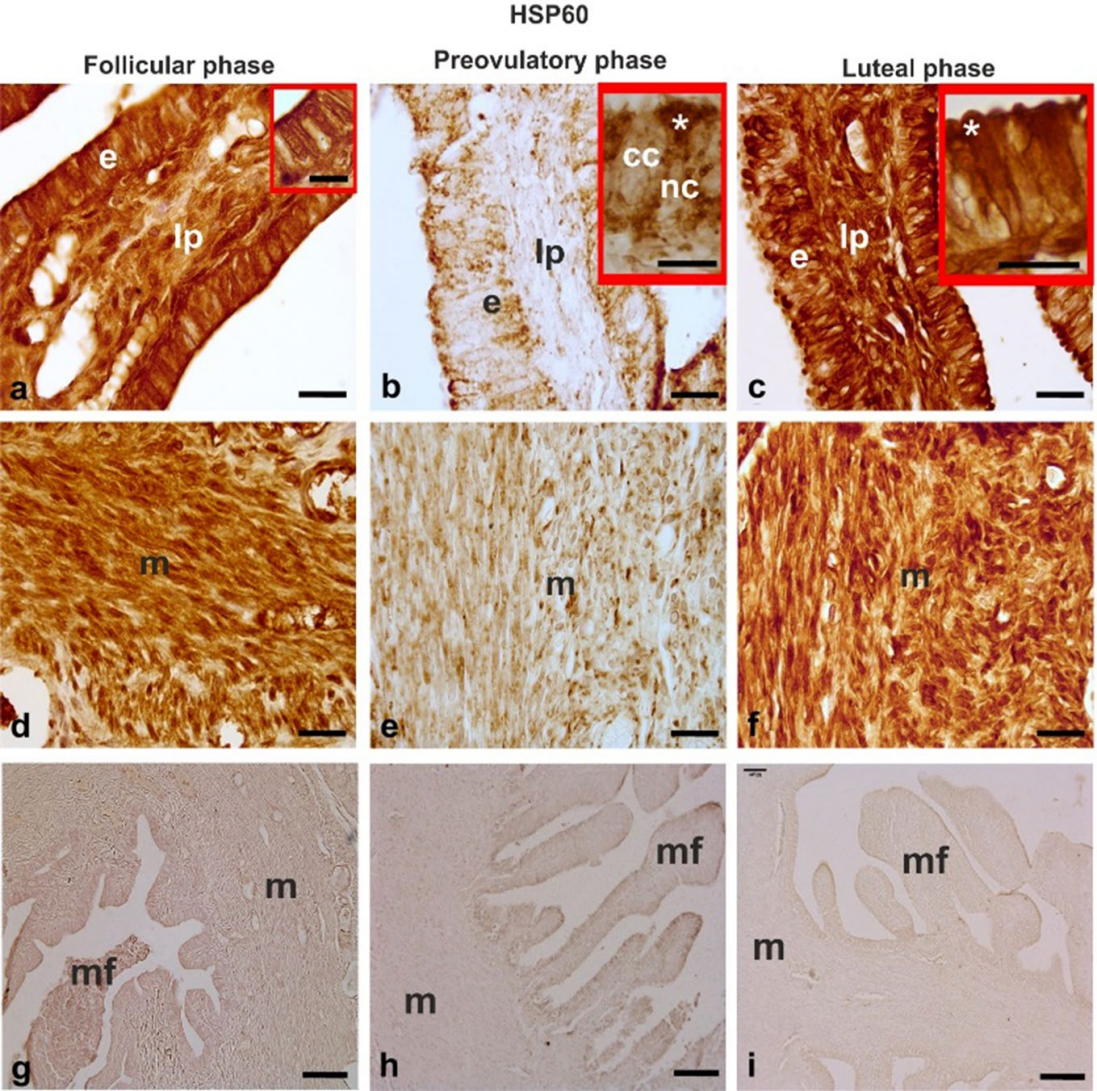
HSP60 immunoreactivity in the baboon (*Papio hamadryas)* oviductal ampulla during the menstrual cycle. The immunohistochemical stain used diaminobenzidine as the chromogen. **a,b,c** show the immunoreactivity in the epithelium and lamina propria of the mucosal folds. The insets of a,b,c show details of the epithelium immunoreactivity. **d,e,f** display the immunostaining of the muscle layer. Note the lower immunostaining in the mucosal fold and the muscle layer of the preovulatory phase compared to the follicular and luteal phases. **g,h,i** show the absence of immunostaining in the negative control procedures. cc, ciliated cell; e, epithelium; lp, lamina propria; m, muscle layer; mf, mucosal fold nc, non-ciliated cells; asterisk, apical region of nonciliated cells. Scale bars: ab,c,d,e,f = 20 μm; g,h,i = 100 μm; insets of a,b,c = 10 μm

**Fig. 4:**
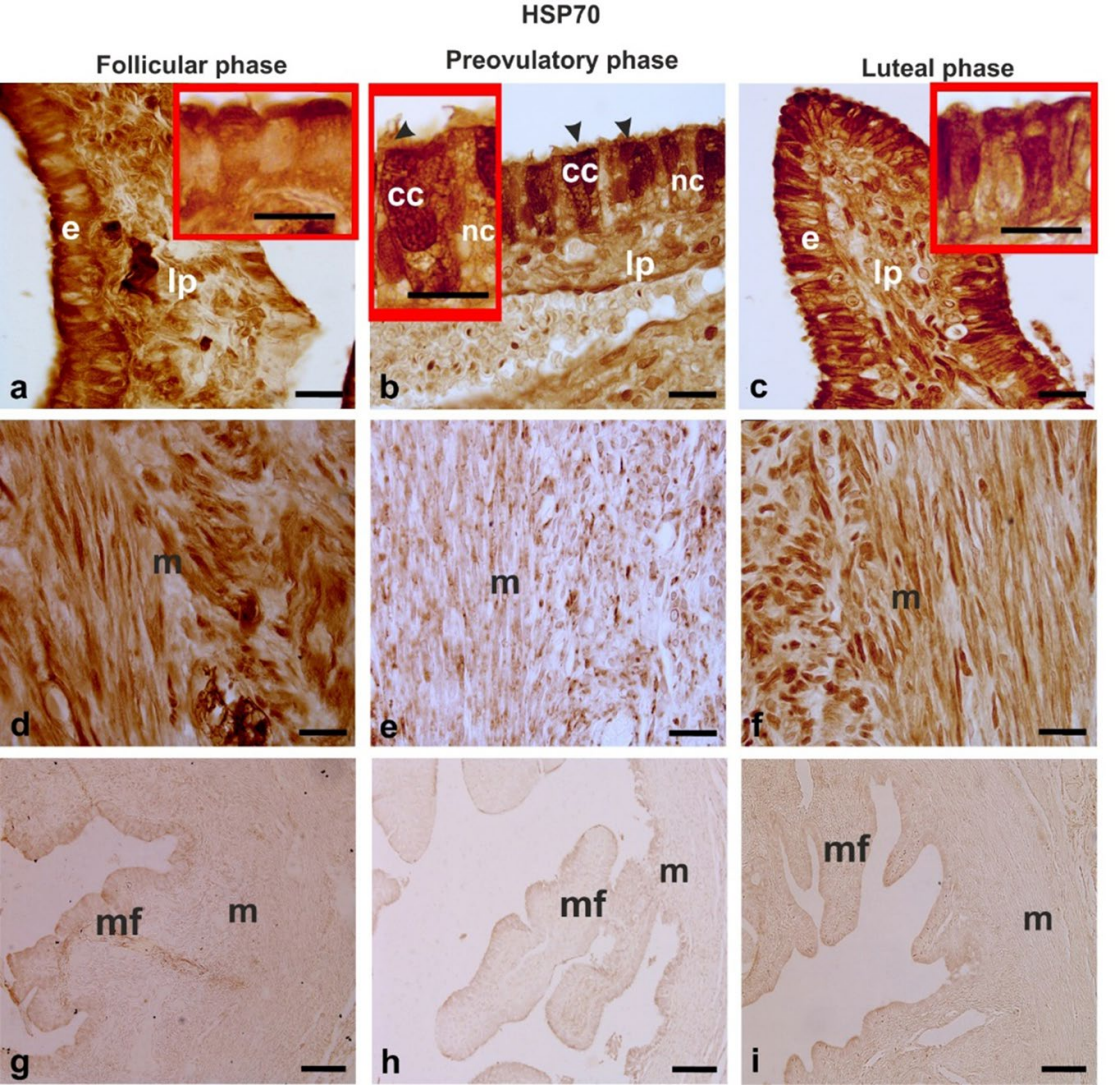
HSP70 immunoreactivity in the baboon (*Papio hamadryas)* oviductal ampulla during the menstrual cycle. The immunohistochemical stain used diaminobenzidine as the chromogen. **a,b,c** show the immunoreactivity in the epithelium and lamina propria of the mucosal folds. The insets of a,b,c show details of the epithelium immunoreactivity. Note in picture b the stronger staining of ciliated cells compared to nonciliated cells during the preovulatory phase. **d,e,f** display the immunostaining of the muscle layer. Note the lower immunostaining in the muscle layer of the preovulatory phase compared to follicular and luteal phases. **g,h,i** show the absence of immunostaining in the negative control procedures. cc, ciliated cell; e, epithelium; lp, lamina propria; m, muscle layer; mf, mucosal fold; nc, non-ciliated cells; arrowhead, cilia. Scale bars: ab,c,d,e,f = 20 μm; g,h,i = 100 μm; insets of a,b,c = 10 μm

**Fig. 5:**
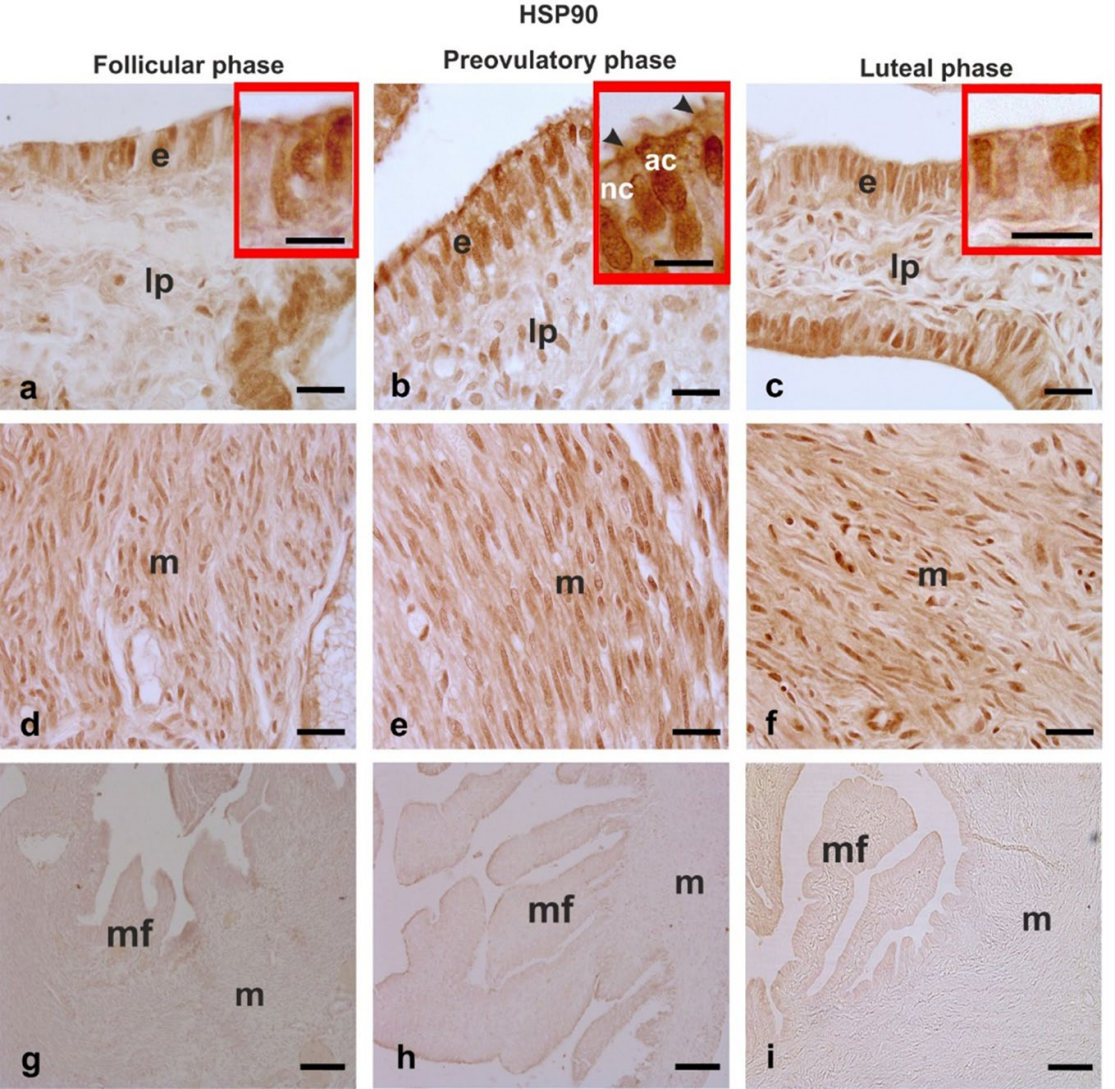
HSP90 immunoreactivity in the baboon (*Papio hamadryas)* oviductal ampulla during the menstrual cycle. The immunohistochemical stain used diaminobenzidine as the chromogen. **a,b,c** show the immunoreactivity in the epithelium and lamina propria of the mucosal folds. The insets of a,b,c show details of the epithelium immunoreactivity. Note the strong positivity of nonciliated cell apical zone and the weak staining of the cilia during the preovulatory phase (inset of b). **d,e,f** display the immunostaining of the muscle layer. Note the stronger immunostaining of muscle cells in the preovulatory and luteal phases compared to the follicular phase. **g,h,i** show the absence of immunostaining in the negative control procedures. ac, apical cytoplasm; e, epithelium; lp, lamina propria; m, muscle layer; mf, mucosal fold; nc, non-ciliated cells; arrowhead, cilia. Scale bars: ab,c,d,e,f = 20 μm; g,h,i = 100 μm; insets of a,b,c = 10 μm

The immunolocalization of HSP60, 70, and 90 patterns in the ampulla displayed variable staining during the menstrual cycle. The results of the immunohistochemical patterns in the mucosal epithelium are summarized in Table 1; Figs. 3, 4 and 5.

**Table 1:** Expression of the HSP 60,70,90 in the mucosal epithelium of baboon *Papio hamadryas* oviductal ampulla during the menstrual cycle

Menstrual phases	Heat Shock Proteins (HSPs)		
	HSP60	HSP70	HSP90
Follicular phase	+/+n/++as	+/+n/++ac	+*/+*
Preovulatory phase	+gNC/++acNC	±NC/++CC/+ci	+n/+acNC/±ci
Luteal phase	++/++n/++ac	++*/++n	+*/+n

ac, apical cytoplasm; as, apical surface; CC, ciliated cells; ci, cilia; g, granular staining; n, nucleus; NC, nonciliated cells

* positivity not in all cells. If not specified, the positivity was detected in both nonciliated and ciliated cells

Results are expressed by a subjective scale: -, negative reaction; ±, +, ++, faintly, weakly, clearly visible immunostainings

#### HSP60 (Fig. 3)

During the follicular phase, HSP60 positivity was observed in the nucleus and the entire cytoplasm of the epithelial cells. Particularly, the epithelium apical surface was strongly stained (Fig. 3a). Also, the stromal cells (Fig. 3a) and the muscle cells (Fig. 3d) showed cytoplasmic HSP60-immunoreactivity. During the preovulatory phase, immunoreactivity was detected in the cytoplasm of the nonciliated cells (Fig. 3b). The immunostaining had a granular pattern and was mainly observed in the apical cytoplasm (inset of Fig. 3b). Also, the stromal cells (Fig. 3b) and muscle cells (Fig. 3e) displayed very weak immunostaining. The luteal phase showed an HSP60 immunostaining pattern like the follicular phase (Fig. 3c,f).

#### HSP70 (Fig. 4)

In the ampulla, the mucosal epithelial cells, stromal, and smooth muscle cells displayed nuclear and cytoplasmic immunostaining for HSP70. During the follicular phase, the cytoplasmic reaction was concentrated in the apical region of the epithelial cells (Fig. 4a). In addition, the stromal and muscle cells were strongly immunostained (Fig. 4a,d). In the ampullae of the preovulatory phase, HSP70 was detected in the cytoplasm of the nonciliated and, to a major extent, in ciliated cell cytoplasm, nucleus, and cilia (Fig. 4b). The stromal and especially the muscle cells were not uniformly labeled and both the cell types displayed immunopositivity ranging from negative to strong staining (Fig. 4b,e). During the luteal phase, the mucosal epithelium showed strong nuclear and cytoplasmic HSP70-immunopositivity (Fig. 4c), also detected in the stromal cells (Fig. 4c) and muscle cells (Fig. 4f).

#### HSP90 (Fig. 5)

In the follicular phase ampullae a well-visible HSP90 immunostaining was observed in the cytoplasm and nucleus of some cells constituting the mucosal epithelium while faint immunoreactivity was detected in the lamina propria (nucleus of stromal cells) (Fig. 5a) and in almost all muscle cells of muscularis (Fig. 5d). During the preovulatory phase, HSP90-positive immunoreaction was localized in the nucleus of all epithelial cell types and the apical cytoplasm of nonciliated cells and in the cilia of ciliated cells (Fig. 5b). No significant change in the intensity and immunostaining pattern of lamina propria and muscularis was observed compared to the follicular phase (Fig. 5e). The ampullae of the luteal phase showed an HSP90-immunolocalization pattern like the follicular phase in the mucosal epithelium (compare Fig. 5c with Fig. 5a), whereas lamina propria and muscle cells displayed slightly higher HSP90 immunoreactivity than the follicular phase (compare Fig. 5c,f with Fig. 5a,d) and the preovulatory phase (compare Fig. c,f with Fig. 5b,e).

## Discussion

Human reproduction has benefited significantly by investigating non-human primate models such as macaques and baboons (Bauer 2015). Their menstrual cycle closely resembles that of women (Goncharov et al. 1976) as well as the estrogen synthesis during pregnancy (Kling et al. 1972) so that the limit deriving from the use of most laboratory animals, whose estrous cycle is strikingly different, is overcome (Brenner and West 1975). Until now, few reports exist on the molecular expression of the baboon oviduct proteins. This work, which planned to enlarge knowledge on the baboon *Papio hamadryas* oviduct, demonstrated for the first time that the abundance and immunolocalization of the HSP proteins are related to the menstrual cycle, which is regulated by the fluctuation of the sex steroids.

The Western blot analysis results showed that the abundance of HSP70 protein was more than HSP60 and 90. Notably, in the luteal phase, the quantitative values of the three investigated HSPs were significantly lower than in the other phases of the menstrual cycle. These findings agree with previous observations (Soleihavoup et al. 2016), which reported a strong reduction of HSP71 (belonging to the HSP70 family) in the luteal phase of the ewe oviduct. The relationship between sex steroids and HSP expression has been observed in other mammals, such as rats (Olazabal et al. 1992) and buffalos (Kalyanaraman et al. 2022), and reinforces what is evidenced by the sex steroid regulation of the HSPs on the oviductal secretome (Binelli et al. 2008).

Also the immunohistochemical analysis results demonstrated changes in the localization and expression of the HSP60,70, and 90 in the ampulla during the phases of the menstrual cycle. As for the ampulla epithelium, HSP60, in addition to the cytoplasmic localization, was unexpectedly found in the nuclei during the follicular and luteal phases, when the nonciliated and the ciliated cells are not distinguishable. The presence of this chaperonine in the cytoplasm and mitochondria of normal cells is well- known (Malik and Lone 2021; Hu et al. 2022). In addition, the nuclear expression of HSP60 has been detected in the nucleus of the oviductal epithelium in women with severe salpingeal pathology such as *Chlamydia trichomonas*-induced oviduct sterility (Zhao and Li 2004).

Interestingly, the present study revealed that during the preovulatory phase, when the nonciliated and ciliated cells were well differentiated, apical positivity was clearly visible in the nonciliated cells. It has been reported that HSP60 is secreted by epithelial cells on the external side of the cell environment (Sangiorgi et al. 2017). Thus, these results suggest that the apical expression of HSP60 is directly correlated to the estradiol level and that HSP60 may constitute part of the material secreted from the nonciliated cells. The ampulla is the site where sperm interaction with cumulus–oocyte complex occurs and where complex physical and chemical factors could exist and contribute to regulating sperm activities. The presence of HSP60 in the apical cytoplasm has been immunohistochemically demonstrated in bovine during the early estrus (Boilard et al. 2004) and in human ampulla (Lachance et al. 2007). In both these species, HSP60 binds to spermatozoa (Boilard et al. 2004; Lachance et al. 2007) without affecting sperm viability, motility, or acrosomal integrity. In addition, a role in the protective integrity of the bovine sperm plasma membrane and in acquiring their fertilizing capacity has been proposed (Lachance et al. 2007). As for the ciliated cells, the present study did not detect the presence of HSP60 in this epithelial cell type. In contrast, Liman (2023) has recently demonstrated the cilia of the ciliated cells of efferent ductules in the domestic cat exhibited strong HSPD1/HSP60 immunostaining.

As for the HSP70, this protein was characteristically much more expressed in the epithelium apical zone during the follicular phase than in the other menstrual phases. We cannot give an interpretation of this finding because previous ultrastructural studies reported that during the follicular phase, the apical region of the epithelium lining the lumen of the baboon ampulla lacks cytoplasmic organelles and secretory granules (Verhage et al. 1997). During the preovulatory phase, HSP70 was detected weakly expressed in the cytoplasm of nonciliated cells and, to a major extent in the ciliated cells and in the cilia. These results align with previous reports on the HSP70 cellular localization (Hu et al. 2022 for references). This study demonstrated that also the expression of HSP70 is responsive to hormonal changes. In rats, the cytoplasm of ampulla epithelial cells shows a different expression of HSP70 during the estrus cycle reaching the highest level during diestrus (Mariani et al. 2000). The involvement of HSP70 in female reproductive functions has been reported in mice (Swelum et al. 2021) because supplementation of the culture medium with monoclonal antibodies against HSP70 inhibits fertilization. As regards the expression of HSP70 in the cilia, our results agree with the evidence of the presence of this protein in cilia or flagella of various organisms such as *Chlamydomonas*, sea urchins, tetrahymena, and rabbits (Williams and Nelsen 1997; Stephens and Lemieux 1999). In cilia, HSP70 is related to axonemal protein dynamics (Stephens and Lemieux 1999). The observed presence of HSP70 in the nuclei of epithelial cells during the follicular and luteal phases is difficult to interpret, although a selective transfer of HSP70 from the cytoplasm to the nucleus has been detected (Mehlen and Arrigo 1994). It has been reported that the nuclear localization of HSP70 has a role in inhibiting apoptosis (Schoedel et al. 2021) and in the heat shock response in vitiligo (Abdou et al. 2013). Long-term exposure of bovine oviduct epithelial cells to high temperature (41 °C) increased the expression of HSP70 with no effect on cell viability, suggesting its putative protective role (Rapala et al. 2018). In addition, it has been suggested that high levels of HPS70 mRNA are related to the rise in the body temperature with a concomitant increase in the temperature of the reproductive organs, which is a common feature in farm animals before estrus and until ovulation (Kalyanaraman et al. 2022). It must take into account that sex steroid fluctuation is responsible for the rise in the basal temperature, which induces the presence of heat stress mediators in reproductive organs during the preovulatory periods (Olazabal et al. 1992; Soleihavoup et al. 2016). In buffalo (Kalyanaraman et al. 2022) and bovine (Bauersachs et al. 2004) oviducts, the maximal expression of HSP70 mRNA has been detected during the late estrous cycle phase. Moreover, an increase in the level of HSP70 in the bovine oviduct has been reported during the late proestrus and estrus phases when the down-regulation of estrogen receptors occurs (Salvetti et al. 2008).

The presence of HSP90 was characteristically detected in the apical region of secretory nonciliated cells and in the cilia during the preovulatory phase. The secretion of HSP90 from nonciliated cells could be implicated in the lumen milieu, whereas ciliary HSP90 could be involved in the ciliary beating and transport of gametes. The presence of HSP90 has been immunohistochemically detected in the cilia of mouse oviducts in which their involvement in the stability of ciliary tubulin and in the regulation of ciliary beating has been suggested (Takaki et al. 2007). This suggests that the expression of HSP90 is important for the progression of spermatozoa along the ampulla. In the oviduct ampulla, diverse sperm movements and activities have been observed in vivo in the mouse model, where complex physical and chemical factors could exist and contribute to regulating sperm activities (Wang and Larina 2023). Since the ampulla is the site of fertilization, it is possible to suppose that HSP90 has a role in this process. A similar trend of HSP90 detected in the present study has been observed in the sheep oviduct (Soleihavoup et al. 2016). These findings meet with the view that HSP90 regulates estrogen receptor signaling (Suuronen et al. 2008) and consequently the morpho-functional features of estrogen tissue target.

The stromal cells (fibroblasts and macrophages) of the baboon oviductal ampulla showed a weaker cytoplasmic HSP60-positivity during the preovulatory phase compared to the other phases of the menstrual cycle. Although previous studies have detected the presence of HSP60 in the ampulla stromal cells of humans (Zhao and Li 2004) and estrus bovine (Boilard et al. 2004), the present study demonstrates the relationship between sex hormone fluctuation and the expression of HSP60 in the oviductal stromal cells of the baboons. Unlike HSP60, the expression of HSP70 in the stromal cells did not show significant changes during the menstrual cycle. To our knowledge, studies on the expression of this chaperonine in the oviduct of other mammals are lacking. However, it has been reported the increased expression of HSP70 in endometrial stromal cells during postpartum in rats (Liman 2017) as well as the role of estradiol in the increased HSP70 in the endometrium of pregnant sheep (Jee et al. 2021). Moreover, the present study revealed the nuclear expression of HSP90 in the ampulla stromal cells throughout the menstrual cycle. No data on the presence of this chaperonine in the oviduct of other mammalian species have been published to date. As for the female reproductive tract, the immunolocalization of HSP90 has been observed in the stromal cells of the human uterus throughout the menstrual cycle (Komatsu et al. 1997) and in postpartum uterus of rats (Liman 2017). Although the functions of HSPs in fibroblasts are unknown, it has been suggested that the expression of HSP70 and HSP90 could be associated with their survival and mobility (Liman 2017).

The smooth muscle cells of the muscular wall expressed cytoplasmic HSP60 immunoreactivity in the ampulla throughout the menstrual cycle, with the weakest immunostaining during the preovulatory phase. To the best of the authors’ knowledge, there is no study illustrating the expression of HSP60 in the oviductal muscle cells of other mammals.

The presence of HSP70 has been observed in the oviduct of rats, although no differences between the segments and the phases of the estrous cycle are reported (Mariani et al. 2000). Our study aligns with data reported in humans by Komatsu et al. (1997); in fact, the baboon myocytes displayed cytoplasmic and nuclear HSP70 immunostaining during the entire menstrual cycle especially during the follicular phase. It has been reported that HSP70 binds progesterone receptors (Smith and Toft 1993) that are highly expressed in myometrial smooth muscle cells during the proliferative phase, and are maintained in the secretory phase (Lessey et al. 1988; Kawaguchi et al. 1991). For this reason, the involvement of this chaperonine in addition to the progesterone in the proliferative activity (Kawaguchi et al. 1991) and in the morphological and functional differentiation of the smooth muscle cells in the menstrual cycle and during pregnancy (Fujii et al. 1990) can be assumed. As for HSP90, in the present study, this protein was found in the nucleus of the muscle cells of the ampulla muscularis throughout the menstrual cycle with a strong expression in the preovulatory and luteal phases. The immunostaining was stronger in the preovulatory and luteal phases than in the follicular phase. There are no studies illustrating the expression of HSP90 in the muscle cells of the oviduct muscularis in other mammals. However, the presence of HSP90 has been observed in the nucleus and cytoplasm of myocytes of rat postpartum myometrium (Liman 2017). HSP90 immunoreactivity has been found in the myometrial smooth muscle cells of women throughout the menstrual cycle and the staining intensity was stronger in the proliferative phase than in the secretory phase (Komatsu et al. 1997). In the latter phase, the expression of estrogen receptors is down-regulated, whereas progesterone is maintained (Lessey et al. 1988; Kawaguchi et al. 1991). It has been reported that HSP90 masks the functional domains to maintain an inactive state (Smith and Toft 1993), as well as it is necessary for the maintenance of the appropriate conformation required for the hormone-binding activity of the steroid receptors (Bresnick et al. 1989). Therefore, the expression of HSP90 in the muscularis of the baboon oviductal ampulla may be related to the change in sex steroid receptor levels during the menstrual cycle. It is known that steroid hormones play a roles in the regulation of ampulla muscular activities that drive the oviductal fluid flow (Barton et al. 2020) and that the HSPs expressed in the smooth muscles are important modulators of muscle contraction, cell migration, and cell survival (Salinthone et al. 2008).

## Conclusions

This is the first report demonstrating the presence of HSPs, namely HSP60, 70, and 90, in the oviductal ampulla of non-human primates such as baboon *Papio hamadryas*. Particularly, a spatial-temporal specificity of the expression of these proteins was observed during the phases of the menstrual cycle. The results strengthen the idea that the expression of HSPs in the *Papio hamadryas* oviduct might be essential for the modulation of the role played by the oviductal ampulla. Although the specific roles played by oviductal HSPs in the oviduct are not well understood, our results contribute to highlighting the indisputable role and the relationship among HSPs, sex hormones, and functional activity in the baboon oviduct. This study provides new insights into the molecular composition of the oviduct of a primate phylogenetically close to humans whose female reproductive apparatus is regulated by the same hormonal events, but in which an extensive study of the expression of HSPs has not been carried out yet.

## Data Availability

All data are available in the present manuscript.

## References

[CR1] Abdou AG, Maraee AH, Reyad W (2013). Immunohistochemical expression of heat shock protein 70 in vitiligo. Ann Diagn Pathol.

[CR2] Abe H (1996). The mammalian oviductal epithelium: regional variations in cytological and functional aspects of the oviductal secretory cells. Histol Histopathol.

[CR3] Barton BE, Herrera GG, Anamthathmakula P, Rock KJ, Willie AM, Harris EA (2020). Roles of steroid hormones in oviductal function. Reproduction.

[CR4] Bauer C (2015). The baboon (Papio sp.) as a model for female reproduction studies. Contraception.

[CR5] Bauersachs S, Rehfeld S, Ulbrich SE, Mallok S, Prelle K, Wenigerkind H (2004). Monitoring gene expression changes in bovine oviduct epithelial cells during the oestrous cycle. J Mol Endocrinol.

[CR6] Binelli M, Gonella-Diaza AM, Silveira Mesquita M, BertanMembrive CM (2008). Sex steroid-mediated control of oviductal function in cattle. Biology.

[CR7] Boilard M, Reyes-Moreno C, Lachance C, Massicotte L, Bayley JL, Sirard MA, Leclerc P (2004). Localizations of the chaperone proteins GRP78 and HSP60 on the luminal surface of bovine oviductal epithelial cells and their association with spermatozoa. Biol Reprod.

[CR8] Brenner RM, West NB (1975). Hormonal regulation of the reproductive tract in female mammals. Annu Rev Physiol.

[CR9] Bresnick EH, Dalman FC, Sanchez ER, Pratt WB (1989). Evidence that the 90-kDa heat shock protein is necessary for the steroid binding conformation of the L cell glucocorticoid receptor. J Biol Chem.

[CR10] Calderwood SK, Mambula SS, Gray PJ, Theriault JR (2007). Extracellular heat shock proteins in cell signaling. FEBS Lett.

[CR12] Desantis S, Ventriglia G, Zizza S, Guaricci AC, Losurdo M, Zarrilli A, Albrizio M (2010). Changes in the expression of the µ-opioid receptor in the mare oviduct during oestrus and anoestrus. Anim Reprod Sci.

[CR11] Desantis S, Albrizio M, Lacitignola L, Laricchiuta P, Cinone M (2022). Modification of morphology and glycan pattern of the oviductal epithelium of baboon *Papio hamadryas* during the menstrual cycle. Animals.

[CR13] Elliott RM, Lloyd RE, Fazeli A, Sostaric E, Georgiou AS, Satake N (2009). Effects of HSPA8, an evolutionarily conserved oviductal protein, on boar and bull spermatozoa. Reproduction.

[CR14] Fujii S, Konishi I, Katabuchi H, Okamura H, Motta PM (1990). Ultrastructure of smooth muscle tissue in the female reproductive tract: uterus and oviduct. Ultrastructure of smooth muscle.

[CR15] Goncharov N, Aso T, Cekan Z, Pachalia N, Diczfalusy E (1976). Hormonal changes during the menstrual cycle of the baboon (*Papio hamadryas*). Acta Endocrinol.

[CR16] Hu C, Yang J, Qi Z, Wu H, Wang B, Zou F (2022). Heat shock proteins: Biological functions, pathological roles, and therapeutic opportunities. Med Comm.

[CR17] Jee B, Dhar R, Singh S, Karmakar S (2021). Heat shock proteins and their role in pregnancy: redefining the function of old rum in a new bottle. Front Cell Develop Biol.

[CR18] Kalyanaraman B, Soundarapandian S, Gopal DR, Sivasankaran B, Manoharan P (2022). Expression profiling of candidate embryotrophic genes of buffalo oviduct during different stages of oestrous cycle. Buffalo Bull.

[CR19] Kawaguchi K, Fujii S, Konishi I, Iwai T, Nanbu Y, Nonogaki H, Ishikawa Y, Mori T (1991). Immunohistochemical analysis of oestrogen receptors, progesterone receptors and Ki-67 in leiomyoma and myometrium during the menstrual cycle and pregnancy. Virchows Arch A.

[CR20] Khalil AA, Kabapy NF, Deraz SF, Smith C (2011). Heat shock proteins in oncology: diagnostic biomarkers or therapeutic targets?. Biochim Biophys Acta.

[CR21] Kling OR, Rubin EJ, Townsley JD (1972). Estrogen synthesis by the pregnant baboon. J Med Primatol.

[CR22] Komatsu T, Konishi I, Fukumoto M, Nambu K, Koshiyama M, Mandai M (1997). Messenger ribonucleic acid expression of heat shock proteins HSP70 and HSP90 in human endometrium and myometrium during the menstrual cycle. J Clin Endocr Metabol.

[CR23] Kopecek P, Altmannova K, Weigl E (2001). Stress proteins: nomenclature, division and functions. Biomed Papers.

[CR24] Kuhn DJ, Zeger EL, Orlowski RZ (2006). Proteosome inhibitors and modulators of heat shock protein function. Update Cancer Ther.

[CR25] Lachance C, Bailey LJ, Leclerc P (2007). Expression of HSp60 and Grp78 in the human endometrium and oviduct, and their effect on sperm functions. Hum Reprod.

[CR26] Lacitignola L, Laricchiuta P, Imperante A, Acquafredda C, Stabile M, Staffieri F (2022). Laparoscopic salpingectomy in *Papio hamadryas* for birth control in captivity. Vet Surg.

[CR27] Lamy J, Labas V, Harichaux G, Tsikis G, Mermillod P, Saint-Dizier M (2016). Regulation of the bovine oviductal fluid proteome. Reproduction.

[CR28] Lessey BA, Killam AP, Metzger DA, Haney AF, Greene GL, McCarty KS (1988). Immunohistochemical analysis of human uterine estrogen and progesterone receptors throughout the menstrual cycle. J Clin Endocrinol Metab.

[CR29] Li S, Winuthayanon W (2017). Oviduct: roles in fertilization and early embryo development. J Endocrinol.

[CR30] Liman N (2017). Heat shock proteins (HSP)-60, -70, -90, and 105 display variable spatial and temporal immunolocalization patterns in the involuting rat uterus. Anim Reprod.

[CR31] Liman N (2023). Heat shock proteins are differentially expressed in the domestic cat (*Felis catus*) testis, epididymis, and vas deferens. Microsc Microanal.

[CR32] Malik JA, Lone R (2021). Heat shock proteins with an emphasis on HSP 60. Mol Biol Rep.

[CR33] Mariani ML, Souto M, Fanelli MA, Ciocca DR (2000). Constitutive expression of heat shock proteins hsp25 and hsp70 in the rat oviduct during neonatal development, the oestrous cycle and early pregnancy. J Reprod Fertil.

[CR34] Mehlen P, Arrigo AP (1994). The serum-induced phosphorylation of mammalian hsp27 correlates with changes in its intracellular localization and levels of oligomerization. Eur J Biochem.

[CR35] Neuer A, Spandorfer SD, Giraldo P, Dieterle S, Rosenwaks Z, Witkin SS (2000). The role of heat shock proteins in reproduction. Hum Reprod Update.

[CR36] Olazabal UE, Pfaff DW, Mobbs CV (1992). Sex differences in the regulation of heat shock protein 70 kDa and 90 kDa in the rat ventromedial hypothalamus by estrogen. Brain Res.

[CR37] Qian SB, McDonough H, Boellmann F, Cyr DM, Patterson C (2006). CHIP-mediated stress recovery by sequential ubiquitination of substrates and HSP70. Nature.

[CR38] Rapala L, Starzynski RR, Trzeciak PZ, Dabrowski S, Gajewska M, Jurka P, Smolarczyk R, Duszewska AM (2018). Influence of elevated temperature on bovine oviduct epithelial cells (BOECs). PLoS ONE.

[CR39] Ritossa FA (1962). A new puffing pattern induced by a temperature shock and DNP in Drosophila. Experientia.

[CR40] Saint-Dizier M, Schoen J, Chen S, Banliat C, Mermillod P (2020). Composing the early embryonic microenvironment: physiology and regulation of oviductal secretions. Int J Mol Sci.

[CR41] Sakatani M, Yamanaka K, Balboula AZ, Takenouchi N, Takahashi M (2015). Heat stress during in vitro fertilization decreases fertilization success by disrupting anti-polyspermy systems of the oocytes. Mol Reprod Dev.

[CR42] Salinthone S, Tyagi M, Gerthoffer WT (2008). Small heat shock proteins in smooth muscle. Pharmacol Ther.

[CR43] Salvetti NR, Baravalle C, Mira GA, Gimeno EJ, Dallard BE, Rey F (2008). Heat shock protein 70 and sex steroid receptors in the follicular structures of induced ovarian cysts. Reprod Domest Anim.

[CR44] Sangiorgi C, Vallese D, Gnemmi I, Bucchieri F, Balbi B, Brun P (2017). HSP60 activity on human bronchial epithelial cells. Int J Immunopathol Pharmacol.

[CR45] Schmitt E, Gehrmann M, Brunet M, Multhoff G, Garrido C (2007). Intracellular and extracellular functions of heat shock proteins: repercussions in cancer therapy. J Leukoc Biol.

[CR46] Schoedel K, Miller V, Osei-Hwedieh D, Watters R, Duensing A, John I, Chandran U, Chang A, Soman V, Weiss K (2021). Differential expression of angiogenesis markers HSP70, HSP90, VEGF and pERK1/2 in both components of dedifferentiated chondrosarcomas. J Bone Oncol.

[CR47] Sima S, Richter K (2018). Regulation of the Hsp90 system. BBA Mol Cell Res.

[CR48] Sirotkin AV, Bauer M (2011). Heat shock proteins in porcine ovary: synthesis, accumulation and regulation by stress and hormones. Cell Stress Chaperons.

[CR49] Smith DF, Toft DO (1993). Steroid receptors and their associated proteins. Mol Endocrinol.

[CR50] Soleihavoup C, Riou C, Tsikis G, Labas V, Harichaux G, Kohnke P, Reynaud K, de Graaf SP, Gerard N, Druart X (2016). Proteomes of the female genital tract during the estrous cycle. Mol Cell Proteom.

[CR51] Stephens RE, Lemieux NA (1999) Molecular chaperones in cilia and flagella: implications for protein turnover. Cell Motil Cytoskeleton 44:274–283. 10.1002/(SICI)1097-0169(199912)44:4<274::AID-CM5>3.0.CO;2-o 10.1002/(SICI)1097-0169(199912)44:4<274::AID-CM5>3.0.CO;2-O 10602256

[CR52] Suuronen T, Ojala J, Hyttinen JMT, Kaarniranta K, Thornell A, Kyrylenko S, Salminen A (2008). Regulation of ERα signaling pathway in neuronal HN10 cells: role of protein acetylation and Hsp90. Neurochem Res.

[CR53] Swelum AAA, Hashem NM, Abo-Ahmed AI, Abd El-Hack ME, Abdo M (2021) The role of heat shock proteins in reproductive functions. In: Asea AAA, Kaur P (ed) Heat shock proteins in inflammatory diseases, Springer Cham vol 22, pp 407–427

[CR54] Takaki E, Fujimoto M, Nakahari T, Yonemura S, Miyata Y, Hayashida N (2007). Heat shock transcription factor 1 is required for maintenance of ciliary beating in mice. J Biol Chem.

[CR56] Verhage HG, Fazleabas AT, Mavrogianis PA, O’Day-Bowman MB, Donnelly KM, Arias EB (1997). The baboon oviduct: characteristics of an oestradiol-dependent oviduct-specific glycoprotein. Hum Reprod Update.

[CR57] Wang S, Larina IV (2023). Dynamics of gametes and embryos in the oviduct: what can in vivo imaging reveal?. Reproduction.

[CR58] Williams NE, Nelsen EM (1997). HSP70 and HSP90 homologs are associated with tubulin in hetero-oligomeric complexes, cilia and the cortex of Tetrahymena. J Cell Sci.

[CR59] Yu H, Hackenbroch L, Meyer FRL, Reiser J, Razzazi-Fazeli E, Nobauer K (2019). Identification of rabbit oviductal fluid proteins involved in pre-fertilization processes by quantitative proteomics. Proteomics.

[CR60] Zhao H, Li H (2004). Immunohistochemical analysis of TNF-α and HSP-60 in women with tubal factor infertility associated with Chlamydia trachomatis. J Huazhong Univ Sci Technol.

